# Australian Community Health and Primary Health Care Policies from 1970s to 2020s: A Policy Analysis of Changing Interpretations

**DOI:** 10.1177/27551938251411284

**Published:** 2026-01-16

**Authors:** Helen van Eyk, Toby Freeman, Connie Musolino, Denise Fry, Colin McDougall, Tony McBride, Virginia Lewis, Fran Baum

**Affiliations:** 1 Stretton Health Equity, 1066 The University of Adelaide, Adelaide, Australia; 2 Sydney Local Health District, Sydney, Australia; 3 Tony McBride & Associates, Melbourne, Australia; 4 2080 Australian Institute for Primary Care and Ageing, La Trobe University, Melbourne, Australia

**Keywords:** primary health care, community health, Australia, health policy

## Abstract

Community health and primary health care (PHC) concepts are contested globally. We analysed 122 Australian federal and state government policy documents relating to women's, Aboriginal community controlled, generalist and workers’ community health services from the 1970s to 2022 using institutional theory to understand how ideas and interests had shaped community health and PHC in Australia. We found an initial flourishing of diverse ideas was contested and constrained over time to reflect a more biomedical, general practice-centric approach to PHC. This was influenced by federal government decisions to prioritise general practice, and state governments’ emphasis on reducing hospital costs. This reflects the power of established institutions and biomedical framings that have constrained debate over what is possible and what practices can be considered. From the content of the policies we reviewed, community health ideas and a comprehensive vision of PHC have persisted in Aboriginal community controlled health services, in women's health policy, and in Victorian community health services. Since pursuing comprehensive PHC remains a vital global goal to improve population health and health equity, our examination of policy history can shed light on how ideas and interests have shaped community health and PHC, and how future policies could be strengthened.

## Introduction

This paper analyses policies relevant to the development of community health services in Australia from the 1970s until 2022. We include policies relating to women's, Aboriginal community controlled, generalist and workers’ health services. The policies analysed are from the Australian federal government, and the governments of the states of New South Wales (NSW), South Australia (SA) and Victoria. These states were selected as the only jurisdictions where community health services persisted beyond the years of the national Community Health Program. Our aim was to investigate the ideas and interests represented in policies that have promoted or discouraged the development of community health services in Australia. We were interested in the extent to which Australian policies reflect the principles and practices of comprehensive primary health care (PHC), as expressed by the World Health Organization's Alma Ata Declaration on Primary Health Care^1^ and elaborated by others.^2–4^ ‘Comprehensive’ PHC includes community participation, responding to local community needs, and intersectoral action on the social determinants of health. It differs from the ‘selective’ approach,^5^ that emphasises a narrower, more technical approach to the biomedical treatment and prevention of disease. Community health and PHC concepts and practices have overlapped and informed each other internationally^6–8^ and in Australia.^9,10^ Fry and Baum define community health services as multidisciplinary teams of salaried health professionals ‘who aim to protect and promote the health of defined communities.’^11^ ^p. 298^

Policy analysis sheds light on framings and interpretations of key concepts and their implications for implementation.^12^ Reflecting on past policies provides valuable insights into how to reform health systems. We derive lessons for contemporary health reform to achieve more embedded, national comprehensive PHC programs, noting that to date, implementation of the comprehensive PHC model globally has been patchy.^13,14^

Health systems are under pressure around the world.^15^ Contributing factors include availability of more expensive technologies and treatments, increasing expectations of health systems, ageing populations, and growing rates of multiple morbidities.^15^ Since 2020 the COVID-19 pandemic has put immense pressure on all levels of health systems and has made visible the inequities in health care access and outcomes within and between countries.^16^ This increasing pressure has led to an international search for health service models that can respond to contemporary challenges.^17^ Strengthening comprehensive PHC has been identified as the most promising option.^18^ PHC has long been recognised as vital to effectively functioning health systems and as the platform for providing basic universal health care and public health services to populations.^19,20^

However, the scope and characteristics of PHC have been contested internationally^21^ and in Australia.^14^ While most proponents of PHC concur that it is the first level of the health system and its organisation should facilitate access, views diverge on three key questions: its scope, how it should be financed, and how to implement it.^21,22^ National responses to these questions have been influenced by social, economic and political contexts.^23^

In recent global discourse, comprehensive PHC has been displaced by an emphasis on universal health coverage.^4,24^ Universal health coverage focuses on the scope, organisation and financing of health services.^25^ The 2018 Astana Declaration on Primary Health Care, released on the 40^th^ anniversary of the Alma Ata Declaration, indicates a primary focus on the achievement of universal health coverage, and the role of PHC in contributing to that goal. Not all have agreed with this formulation.^24,26^ Sanders et al,^4^ ^p. 620^ argued ‘By reducing PHC to a cornerstone of UHC [Universal health coverage], as opposed to an umbrella under which UHC resides, the Declaration of Astana confines the health sector to a much more restricted role*’* with insufficient attention to social determinants of health and community participation.

Like many high income countries, community health in Australia has failed to flourish and has been marginalised by a dominant focus on biomedical services, particularly acute care.^14^ As a contribution towards understanding the forces shaping this outcome, we used institutional theory from political science^27,28^ to investigate how the meanings of community health and PHC have been represented in Australian written policy.

### The Australian Context

Australia is a federated nation with six state and two territory governments (henceforth called state governments). It has a complex mix of services and health care providers, funded by Australian federal and state governments, the not-for-profit and private for-profit sectors; and individuals.^29,30^ The federal and state governments have shared responsibilities for health care and roles including funding, policy development, commissioning, delivering, and regulating the health system. The not-for-profit and private for-profit sectors also operate private hospitals and the latter pharmacies and medical practices, and there is a large private health insurance industry. Medicare, Australia's universal health care insurance scheme, is funded by the federal government through taxation revenue and a levy, and funds access for all citizens to hospital and medical services, allied health services, and testing and imaging, albeit with a co-payment for most services except public hospital care. Medicare is the underpinning funding system for general and specialist medical practice, and public hospitals.

Prior to the 1973 Community Health Program, the health system was overly reliant on the private sector, creating financial access barriers, and was largely focused on curative services, with little capacity for prevention and health promotion.^9^ While there were some pioneering community health services in Australia prior to 1973 including Aboriginal health services, the national Community Health Program introduced in that year by the Whitlam Labor government seeded community health services nationwide. However, the program was short lived. A change of government in 1975 led to reductions in and later abolition of identifiable federal community health grants to the states.

Despite the loss of federal funding, state governments in Australia have funded or directly operated a variety of community health services to different extents, including services for children, young people, migrants, women, and Aboriginal and Torres Strait Islander people, that complement mainstream general practice, and often focus on equitable access to PHC. Community health services are mainly funded by state governments and run by government and non-profit organisations, and are diverse in terms of service mix, organisation, size and management structure. They include comprehensive PHC-oriented services (such as health promotion, maternal and child health services, and in some cases primary medical care) and services such as community mental health, home nursing and chronic disease management, parts of which can be seen as pre and/or post-acute services.^31^ Service differences within and between states have resulted in a patchwork system, complicating coordination but also potentially stimulating variety and innovation.

## Methods

This policy research is part of a larger Australian Research Council Discovery research project on the history of community health in Australia. Ethics approval was received from the Flinders University Human Research Ethics Committee (project #4168).

### Policy Selection Process

We collected 122 Australian, NSW, SA and Victorian community health policy documents from the 1970s onwards, using library, internet and archive searches. These states were selected because they showed the strongest implementation of community health historically outside of the Aboriginal Community Controlled Health Organisation (ACCHO) sector.

To be included, documents needed to include goals, objectives and strategies relevant to community health. We included policy documents related to general community health, including PHC, and to Aboriginal health and women's health as these are all underpinned by a comprehensive vision of PHC. Policy documents included formal government policies, and discussion papers and reports that influenced government decisions and action. We did not include operational policies, guideline documents or technical papers. The research team included investigators with long-term experience and knowledge from each of the case study jurisdictions who advised on and assisted in identifying relevant strategic policy documents. Analysis was conducted on the full set of available policies (n=122). A full list of the policy documents is available in supplemental file 1.

### Document Coding and Analysis

The research team undertook collaborative thematic analysis of the documents^32^ using NVivo qualitative analysis software. We undertook a theoretical thematic analysis to identify semantic and latent themes in the policy document,^32^ informed by a critical realist standpoint to understand the meanings and ideas inherent in policy representations of community health and primary health care.^33^ We followed Braun and Clarke's^32^ steps for good thematic analysis to ensure rigour. A coding framework was created based on preliminary review of the policy documents and further developed during team discussions. The codebook incorporated community health and PHC concepts, and related elements such as community development, intersectoral collaboration, social determinants of health, equity, multidisciplinary teams, prevention, and health promotion. This paper analyses community health, PHC and related terms to understand their changing meanings within policy documents over time.

We used institutional theory to inform our analysis. There are different approaches to institutional theory and analysis.^34^ We drew on the work of Scott^28,35^ in particular to understand how organisational fields, in this case the community health sector, are shaped by ideas, actors (individual and organisational) and interests in the field, and by institutional forces that shape the regulatory environment, norms, and ways of working.^35,36^ Scott, an organisational sociologist, defined institutions as ‘social structures… composed of regulative, normative, and cultural-cognitive elements that, together with associated activities and resources, provide stability and meaning to social life’.^28^

Institutional theory has been valuable in health services research to understand the drivers of the factors that have shaped models of health care, and the rise or fall of particular models of health care, including primary health care.^35–39^ Baum and Freeman^14^ identified a dominance of medical ideas on models of care, the powerful interests of private general practice and medical lobby groups, and cost containment and privatisation government values as barriers to the flourishing of community health models in Australia. Javanparast et al^36^ identified that for the Australian government-funded primary health care organisations (Medicare Locals), regulatory barriers, including contracting and funding processes, conflicted with normative forces on organisations to address social determinants of health, resulting in more medical approaches to primary health care. Windle et al^40^ found limited flexibility in funding and ideologically driven framing of health problems shaped the next iteration of Australian primary health care organisations too (Primary Health Networks) . Roussy and colleagues^38,39^ used institutional theory to understand how regulatory forces, neoliberal norms, and a desire to protect ways of working pushed community health services in Victoria, Australia, to merge. Pineault et al^37^ found in Canada that a powerful normative institutional force following health service reform was exemplar health services acting as models of change that other services followed.

We used the content coded in NVivo to interrogate the role of ideas around community health and PHC, and how these shaped the institutional field of community health in Australia over time. The research team discussed the identified themes, and further developed and refined them in analysis workshops. We deepened our data analysis by drawing on the literature and the researchers’ extensive knowledge and experience in community health in Australia.

## Results

Our policy analysis enabled us to track changing representations of community health and PHC in policy. Using institutional analysis, the findings from our analysis are presented through the themes of:

- A flourishing of ideas in community health
- How ideas of ‘community health’ and ‘primary health care’ have been used in Australian policy
- The contest of ideas in community health
- Policy areas where community health ideas have thrived.

### A Flourishing of Ideas in Community Health

In May 1973 the new reforming Labor federal government funded a national Community Health Program, as recommended by the report ‘A Community Health Program for Australia’,^41^ ^p. 4^ which stated:

‘The objective of a national program should be to encourage the provision of high quality, readily accessible, reasonably comprehensive, co-ordinated and efficient health and related welfare services at local, regional, state and national levels. Such services should be developed in consultation with, and where appropriate, the involvement of the community to be served.’

The report stated that services should be provided as integrated comprehensive programs to people in specifically defined regions or localities, without charges. Services should include information and counselling to ‘improve the habits, conditions and environment that may precede disorders of health’,,^41^ ^p. 4^ direct preventive action, disease detection procedures, information for individuals to seek care, specific diagnostic and treatment services, and rehabilitation and help for those with chronic disability. The section on Community Health resources described primary care as: ‘what most people use most of the time for most of their health problems … the point of entry into a comprehensive health system.’,^41^ ^p. 5–6^ It emphasised the basic primary care team as ‘essential’,,^41^ ^p. 6^ and described its three key components as primary medical care, nursing care, and health outreach and social advocacy.

To implement the Program, the federal government funded block grants to state governments, and project grants to non-government organisations and community groups to set up a wide range of community health centres, services and other projects. The policy noted ‘a uniform array of community health resources is not intended.’,^41^ ^p. 6^ Its multiple goals could be and were read ambiguously, giving rise to differing interpretations and practices. This variation was reflected in the diversity of projects funded.^42^ By 1976, the Review of the Community Health Program reported 727 funded projects. They included community health centres in metropolitan and rural areas, women's refuges and health centres, family planning services, specialist training for general practitioners, foundation chairs of Community Medicine in universities and multidisciplinary training.^43^

The Program was a federal policy and funding program, and was implemented mainly via states. Each state had its own ideas, interests and institutional contexts which in turn shaped how the Program was taken up. The three state governments we studied responded differently to the Community Health Program. The NSW government used federal funds to set up programs and services in underserved inner and outer metropolitan and rural areas, and used its state public service structures to employ staff. While consultation with community groups took place, management by local communities did not occur. Primary medical care was excluded, except in rare exceptions which were not sustained. In Victoria, the government was opposed to accepting federal funds to implement what it saw as federal health policy, but community groups were enthusiastic, and successfully used the provisions of the 1958 Victorian Hospital and Charities Act to set up community organisations to receive funds, employ staff and run community health centres with community boards of management.^44^ Some centres included primary medical care funded in a range of ways. The SA government established some centres alongside general medical practices, and others had a greater focus on allied health services, health promotion, and advocacy. Community health staff were employed as public servants and community health centres had community boards of management. Training courses for allied health workers were established, including innovative courses leading to the development of new roles and categories of health worker, such as community nurses.^43^

### How Ideas of ‘Community Health’ and Primary Health Care’ Were Used in Policy

The Community Health Program pre-dated the World Health Organisation's 1978 Alma Ata Declaration on Primary Health Care,^1^ which described principles that matched those of Australian community health as ‘primary health care’. The 1986 Review of the Community Health Program by the Australian Community Health Association, continued to use the term ’community health’, and described these services as ‘publicly funded primary health care’ (ACHA, 1989). The 1994 Towards a National Health Policy Discussion Paper also referred to ‘community health’ (and listed it as a type of primary care service), but by the 2009 A Healthier Future For All Australians Final Report, the terminology had switched to PHC.

Neither the term ‘primary health care’ nor the Alma Ata Declaration were referenced in SA documents prior to 1988, despite earlier papers grappling to define ‘community health’. The switch to the term PHC occurred in 1988 with the ‘Primary Health Care in South Australia’ discussion paper, which referenced the Alma Ata Declaration in defining PHC. This paper argued that:

Community health services are important publicly-funded primary health care services. The initial Community Health Programme for Australia (1973) contained many goals in common with primary health care and while the practice has not always been consistent with these goals there is still considerable accord.,^45^ ^p. 19^

Community health services were subsequently renamed PHC services in SA in the 2000s.

By contrast, Victoria never ceased using community health terminology in its policies or services. The earliest references we found to PHC in Victorian policy was a 2002 discussion paper, where community health was conceptualised as one type of PHC service.^46^

NSW Health combined the two terms in the phrase ‘Primary and Community Health’ ‘to describe the overlapping primary health care and community health sectors and services as a single integrated and cohesive structure.’,^47^ ^p. 1^ Despite the policy, general practice and community health services in NSW remain distinct structures.

### The Contest of Ideas in Community Health

Understandings and framings of community health and PHC have changed and been contested over time (see Table 1).

**Table 1.** Constants and Changing Understanding of Primary Health Care Terminology in Australian Health Policy from 1970s to 2022.

Primary health care terminology	Changes in meaning/ understanding/ emphasis	Constants in meaning/ understanding
Community health	Less used in policy over time, apart from in Victoria where a community health sector exists separate to other community services.	Umbrella term used to differentiate a range of services delivered in community settings by any type of provider.
PHC	Move from comprehensive PHC, including community development, social advocacy, including related welfare services to more clinical services sometimes described as primary care or primary medical care. Move from broad interpretation of PHC to being mainly about GPs.	First point of contact with health system, gatekeeper to acute services. A level of the health system. Importance of general practice. Criticisms of illness focus of health system.
Multidisciplinary team	From inclusion of social work, community development and social advocacy roles with team members as equal partners, to more clinical focus on allied health and nurses supporting general practice.	
Community development	Most apparent in 1980s policies in all jurisdictions, but then not visible again until 2000s in Victorian policy and in the SA Primary Prevention Plan (2011) – subsequently revoked	Continued to mean local community capacity building and empowerment, working with people in communities rather than solely with consumers.
Chronic disease management	Grew in emphasis and priority due to increasing pressure on health system budgets and functioning.	Role of community health (variously defined and funded) in chronic disease management
Health promotion and prevention	Became more structural and policy-oriented recognising impact of social determinants. Focus for prioritisation of health promotion/prevention funding on chronic disease prevention/management.	Ongoing focus on lifestyle and behaviour change, with some exceptions (eg policies/regulation for tobacco control). Calls to prioritise funding of health promotion and prevention.
Integration/ coordination of care	Increase in emphasis as a result of increased focus on chronic disease management.	Role of community health (variously defined and funded) in case management and coordination in complex cases.

PHC has been defined since the 1970s as the first point of contact in the health system, the gateway to acute and specialist health services, and the basis of community health services. The positioning of federal and state PHC policy shifted over time towards a more biomedical and clinical interpretation. By biomedical ideas, we mean ideas in policy that reflect the biomedical approach to health that emphasises the detection and treatment of disease in human bodies, rather than more holistic approaches to social causes of disease, and the consideration of wellbeing beyond the absence of disease.^48^ For example, the 2010 National PHC strategy had a particular emphasis on chronic disease, accounted for within a biomedical framework: ‘Chronic disease is estimated to be responsible for more than 80 per cent of the burden of disease and injury suffered by Australians. More than half of all GP consultations are with people with a chronic condition. Of the estimated 731,000 potentially preventable hospital admissions in 2007–08, around 32 per cent of these were related to complications from diabetes. Better management of chronic disease is essential to reduce the burden on hospital care.’^49^ ^p. 31^ These shifts are described further below and are summarised in a timeline in the Supplemental material.

### The Contest of Ideas in Federal Governments

In Australia since at least the 1960s, there have been two competing interpretations over the scope of PHC and how it should be delivered: as fee for service general medical practice delivered in private practice and subsidised by Medibank and later Medicare; or within community health services as publicly funded salaried multidisciplinary teams focused on defined communities, with no costs to users. Since the introduction of the CHP these two differing conceptions have competed for pre-eminence and government favour with the fee-for-service general practice model winning far more policy attention and funding.

Federal policy priorities from the mid 1980s shifted away from community health ideas. The federal Labor government's health policy from 1983 to 1996 emphasised primary medical care, establishing Medicare in 1983 and the General Practice Strategy in 1991. In the 1990s the institutional context of community health began to be heavily shaped by increasing budgetary and demand pressures on Australian hospitals and health services. There was increasing emphasis on cost effectiveness and the careful targeting of funding, and while the value of a strengthened PHC sector was acknowledged, general practice received the most attention, rather than on a broader PHC approach.^50,51^

The health sector focus of the conservative Howard government (1996-2007) was on the role of the private sector in health care provision, and they instituted a series of incentives for people to take up private health insurance. These federal subsidies for private health insurance cost $6.5 billion in 2024.^52^ Reforms in PHC, which was less affected by these changes, were aimed at improving coordination of care, but there was no overarching PHC policy.^53^

Elected in 2007, the federal Rudd Labor government commenced extensive consultations for national health reforms in 2009. The reform agenda proposed comprehensive PHC centres and services, and PHC organisations to coordinate general practice services and undertake population health planning.^49^ The proposed centres were intended to bring together state funded PHC services and Medicare funded general practice services to focus on health promotion, early intervention and better management of chronic disease. This would have given the federal government full responsibility for funding and policy of all PHC and community health services. The reform agenda was not progressed in the form proposed,^54^ partly due to a change in Prime Minister and a lack of enthusiasm from some state governments. Concern for the increasing burden of chronic disease, and the increasing pressure on hospitals, remained evident in policies, with a resulting focus in PHC policy on chronic disease prevention and management, hospital avoidance and cost containment (eg,^47,55,56^) losing the broader agenda evident in the 1970s and 1980s.

The 2010s saw some broadening of PHC in some Australian policies. The 2013 ‘National primary health care strategic framework’ recognised that PHC is broader than general practice. The policy promoted a ‘consumer focused and integrated primary health care system’ with an increased emphasis on ‘improving access and reducing inequity’, and ‘health promotion and prevention, screening and early intervention’. While it continued to focus on PHC to ‘achieve better health outcomes at a lower cost’ and ‘to ease the burden on hospitals’ the policy also recognised the need for intersectoral partnerships to address social determinants of health, such as housing, education and employment.^57^ ^p. 6^

### The Contest of Ideas in Different States

Across the states we studied, there was a contest of ideas between medicalised care and a social view of health as the basis of PHC, and between the local responsiveness and innovation in the original Community Health Program, and more centralised management. In all three states, there were contradictory moves towards centralised planning and/or regionalisation, commencing in the 1980s in NSW, and in the 1990s for SA and Victoria.^58^ However, the institutional forces and interests played out differently across the different state contexts, discussed in turn below.

#### Victoria

In Victoria, historically community organisations had a large role in health and community services and saw community activism as part of their work.^59,60^ Before 1992, Victorian policy continued a focus on locally managed community health centres, and comprehensive PHC to maintain population health. Victorian community health centres adopted a social view of health, utilising ‘whole of person care’, and ‘noisy advocacy’ to promote the needs of their local communities,^61^ ^p. 158^ maintaining a comprehensive PHC approach. These strengths persisted despite community health funding being only 1.9% of the state health budget.^61^ The Kennett Liberal government (1992-1999) withdrew support for PHC, cutting funding and introducing market-based reforms across government funded services including a competitive approach to contracting services and programs.^62^ This required permanent changes in the structures, governance and scope of services over the next decade as the sector adapted and did its best to continue its role. Following the election of a Labor government in 1999, the ‘Towards a community health policy framework: a discussion paper’ identified the role of ‘primary health care services in contributing to improved health and well-being and reduced demand for more specialised and acute services.’^46^ ^p. 22^ Subsequent policies during the 2000s referred to the role of community health organisations in providing primary health and health promotion services,^63^ that "complement’ federally funded PHC.^64^ ^p. 27^ This document stated that community health centres ‘provide a range of primary healthcare services such as community nursing, allied health, community development, chronic disease management and health promotion services.’^64^ ^p. 27^

In 2020, Victoria's community health sector was described as ‘a critical part of Victoria's healthcare system, providing locally-based, affordable health and social care to disadvantaged Victorians.’^65^ ^p. 1^ The reform plan expected community health centres to work in partnership with ‘primary, acute and social sectors’ (p. 1) and ‘operate GP-led models of care to increase access to affordable primary care’ (p.2).

#### South Australia

SA's strong commitment to community health, comprehensive PHC, and a social view of health continued into the 1990s.

Health Minister Lea Stevens committed pre-election to a comprehensive PHC approach and commissioned the 2003 Generational Health Review. The review promoted community health and PHC services, but a change of health minister and lack of Cabinet support meant this progressive review was never implemented and instead the narrower 2007 ‘GP Plus Health Care Strategy’ was adopted. It provided the policy basis for the establishment of GP Plus Health Care Centres that were intended to complement and increase collaboration with local GP services.

A subsequent review of non-hospital based services^66^ led to the withdrawal of funding for community development and group-based services and concentration on hospital avoidance and chronic disease management. This extreme withdrawal from PHC by the SA government was justified by the Australian government proposal to take full responsibility for funding all PHC services in Australia described above. Most health promotion and non-clinical PHC services across SA were de-funded. The 2011 Primary Prevention Plan, intended to be ‘the next step in the development of primary health care reforms’^67^^p. 1^ and which advocated for comprehensive PHC, was formally withdrawn. These changes in SA policy in the 2000s and 2010s show how political and policy change can rapidly transform the nature of PHC policy and practice.

#### New South Wales

In NSW, public hospitals and community health services within defined geographical areas have been managed jointly by Area Health Services (from 1986) and Local Health Districts (since 2011). This approach has had bipartisan political support. The ‘Integrated Primary and Community Health Policy 2007–2012’ sought to identify commonalities and differences between PHC services, including general practice and community health services. This policy differentiated PHC from community health, describing PHC as ‘universally accessible, generalist services (eg general practice, community/early childhood nursing services) that address the health needs of individuals, families and communities across the life cycle’, and community health services as operating predominantly ‘from a social model of health whereby improvements in health and wellbeing are achieved by directing efforts towards addressing the social and environmental determinants of health.’^47^ ^p. 1^ The ‘HealthOne’ model was proposed which ‘brings together GPs with community health and other health professionals in multidisciplinary teams’,^68^ ^p. 6^ and offered funding for this purpose. However, neither the policy nor the model explained how the physical, structural and funding differences between general practice and community health services could be aligned.

### Policy Areas Where Community Health Ideas Have Thrived

There remain community health principles in Victorian and NSW policies, the Aboriginal community controlled health sector, and to a lesser extent, in women's health policies.

#### Aboriginal Community Controlled Health Organisations (ACCHOs)

ACCHOs were first established in the 1970s^69^ and pre-dated the Alma Ata Declaration. In 2022, there were over 140 ACCHOs in urban, regional, and remote Australia. ACCHOs arose in response to the impacts of colonisation, continuing racism, poor access to mainstream health services, and social and economic inequities.^70,71^ ACCHOs continue to present a model of community health that prioritises accessibility, community participation, action on social determinants of health, and holistic, multidisciplinary health care.^72,73^

Aboriginal health policy nationally and in the states has maintained a comprehensive PHC approach and model of service delivery since the 1970s. The 1989 ‘A National Aboriginal Health Strategy’ was a landmark policy that emphasised the importance of self-determination, self-reliance, recognition of unceded sovereignty, and participation of individuals, families and communities in health services. Since then, Australian federal and state Aboriginal health policy has continued to recognise the unique expertise and success of ACCHOs in the provision of PHC services to Aboriginal and Torres Strait Islander people, and Aboriginal and Torres Strait Islander holistic views of health. Policies have promoted building partnerships between state and federal governments and ACCHOs, cultural awareness in all health services, and the need to strengthen the Aboriginal health workforce.

### Women's Health Policy

Women's health centres in Australia were established with federal CHP funds. The federal government provided support for the first Australian Women's Health Conferences, contributing to policy development. The first Australian Women's Health Policy in 1989 was the first of its kind in the world. Its development was supported by the Australian women's health movement,^74^ and informed by previous SA and Victorian women's health policies.

Women's health policies included models for women's health centres which operated within a PHC framework alongside a feminist philosophy, within the second wave feminist social movement,^75^ and reflected a comprehensive PHC approach. For example, ‘Women's health centres of South Australia: 5 year strategic plan’ stated that women's health centres were:

1. ‘to act as a model for change to ensure that the principles of Social Justice; equity, access and participation, are fully realised within the health system,
2. to promote the participation of women in health decision-making at all levels,
3. to provide alternative, complementary services which integrate clinical services, health education, health promotion and preventive health care.’^76^ ^p. 9^

Women's health policy often prioritised issues such as reproductive health and sexuality, and breast and cervical cancer screening, which can be more clinically focused. However, a social determinants of health approach was also evident, including priorities related to gender inequities, and priorities for particular groups of women, for example Aboriginal women, migrant women, carers, and those experiencing socio-economic disadvantage, and issues such as mental health, housing, and safety. The 1989 National Women's Health Policy defined health broadly, as ‘determined by a broad range of social, environmental, economic and biological factors’, with health status and outcomes differences ‘linked to gender, age, socioeconomic status, ethnicity, disability, location and environment.’^77^ ^p. 5–6^ Similarly, NSW's 2000 ‘Strategic framework to advance women's health’ stated that ‘NSW Health recognises the links between women's social experiences and position within society and their health status, and remains committed to addressing these, and other, social determinants of health’,^78^ ^p. iii^ as evidenced by the continued support for 21 non-government women's health centres funded by NSW Health. These centres are based on a woman-centred approach to PHC. Services include some women specific medical care, ‘preventative health care, community education and development, advocacy and empowerment.’,^79^ ^p. 11^ The Victorian Women's Health Program was established following extensive community consultation published in a report which stated that ‘the health and well-being of women is directly related to the social context in which women live their lives.’^80^ ^p. 1^ A statewide network of women's health centres and services was developed in the late 1980s, and continues today, including three state-wide services and nine regional services focused predominantly on population health promotion, advocacy and research.

## Discussion

Our review of community health and PHC policy over 50 years highlights two crucial points that are relevant to PHC policy in other countries: 1) that PHC (even though differently defined) has continued to be seen as central to a well-functioning health system, and often as the way to reform and improve health systems; and 2) both from within and outside PHC, there have been differing ideas and interests competing over what PHC is and what form it should take. This is because the contest over PHC is not simply a contest of ideas, but a contest for political influence and resources. This dispute means that different models have been promoted at different times and in different jurisdictions, reflecting interests at play and institutional forces in different institutional contexts. Within the policy discourse of PHC, medical power, political imperatives, commercial factors and advocates for salaried multidisciplinary models compete for influence and the contest over definitions reflects this.

Our institutional theory analysis indicates that Australian community health centres and services led to a flourishing of new ideas about what community health was and what it could achieve. Community health policies in the 1970s and 1980s in Australia were consistent with the principles and values of the WHO 1978 Alma Ata Declaration and 1986 Ottawa Charter for Health Promotion, and sought to apply them to the Australian context.^10,14^

Interpretations of PHC changed from comprehensive PHC to individual clinical care, and from a multidisciplinary approach to a more medicalised perspective centred on fee-for-service general practice (see Table 1). While the importance of chronic disease as a population heath issue has been recognised since the 1970s, it is now a primary focus of health policy, along with care coordination.

There was also recognition that medicine and medical technological advances are consuming increasing proportions of the health budget, partly due to a higher proportion of the population with chronic diseases, rising costs of technology and increasing health professional salaries. In response, Australian PHC policy has included strategies to increase practices that only charge the government rate and don't include co-payment, after hours care, and better care coordination.^81^

In Australia, this contest between differing interpretations of PHC for pre-eminence and influence in government policy has been related to:

1. Divided responsibility between federal and state governments for PHC
2. A contest between public and private health sector ideas and interests.
3. A contest between medical and community ideas and interests.

These policy tensions have been present to a greater or lesser degree in policy documents in the last 50 years.

### Divided Responsibility for Health Care Funding and Delivery

The division of responsibilities between federal and state governments has evolved due to a combination of chosen political commitments (eg, federal funding of Medicare) and historical precedent (state responsibility for hospitals and some public health functions). This means neither states nor the federal government have overall responsibility and leadership of the health system. The federal government has superior access to resources through tax collection, but state governments operate hospitals and community health services, necessitating negotiation over funding.

While this divided, and at times contested, responsibility has created difficulties, it has sometimes been advantageous. Both federal and state governments have, at different times, supported innovation and protected comprehensive PHC and community health services, and have also cut funds and withdrawn from policy. Divided responsibility has enabled more diversity in community health services, and the development of regionally and locally responsive approaches. Australia is far from alone in being a federated country with split responsibilities for health care, and these contests of interests can be found in many countries globally.^82^

This division of responsibilities also contributes to very limited knowledge at the federal level of the community health sector, and a lack of attention to its potential contribution to federal policy. Federal government agencies such as the Australian Institute of Health and Welfare and the Productivity Commission briefly mention community health services, but do not include data on their extent, activities or impact, instead focusing on general practice data.^83,84^

### Contest Between Public and Private Health Sector Ideas and Interests

The impact of the contest between the public and private sectors in Australia's health system is arguably more profound. Medicare subsidises fee-for-service private medical practice, and federal policy supports this specific interpretation of PHC. States fund and/or manage salaried multidisciplinary community health services, drawing on a more comprehensive interpretation of PHC. The ongoing if uneven co-existence of these public and private sectors has been a policy choice of both conservative and progressive federal and state governments, continued and consolidated over time; exacerbated, but not caused, by divisions in responsibilities. A neoliberal approach to policy that emphasises individual responsibility for health, a reduced role for governments, new public management approaches, and private provision of services, has been increasingly evident in Australia since the 1980s.^85^ This has had a detrimental effect on community health ideas and funding.^86,87^ The hybrid marketisation model of Medicare is a defining feature of PHC in Australia, which has allowed a form of universal health care to continue under neoliberalism, but has constrained its capacity for transformative system change, fostered a liberal focus on individual choice and competition, and funded a largely private primary medical care sector.^88^

### Contest Between Medical and Community Ideas and Interests

In Australia, medical power has continued to influence policy and funding. From its beginnings the national Community Health Program was criticised by medical lobby groups such as the Australian Medical Association and Royal Australian College of General Practitioners (RACGP), with some advocates arguing community health was ‘third world medicine’.^89^ ^p. iii^ The lack of primary medical care in community health services (with some exceptions) reflects this influence. These groups remain powerful lobbyists representing their professional interests in policy and funding.^90^

Acute care remains dominant with the need to expand hospitals and maintenance of fee-for-service for specialist medicine outside hospital largely unquestioned, while PHC often has to justify its existence and budget allocation through its potential to reduce hospital demand and impact on population health.

Our analysis shows that health policy ideas that support comprehensive PHC, such as equity, a social view of health, community participation, and health promotion and prevention, have continued to show less traction in policy than biomedical and clinical ideas. The government's ongoing lobbying by medical groups such as the RACGP and Divisions of General Practice and their successors have likely reinforced this.

For generalist community health, advocates could not match the resources and influence of established institutions and medical groups, and the dominant political, bureaucratic and popular understandings of health care as primarily about doctors and hospitals. The exceptions to this are the social movement for Aboriginal rights and self-determination from which ACCHOs emerged, and the feminist movements which informed the establishment of women's health services. For these examples, community interests appear to have strongly informed policy and models. These sectors hold learnings for how generalist community health ideas and community interests can better inform policy.

The interaction between these actors, ideas, and interests are summarised in Figure 1.

**Figure 1.**
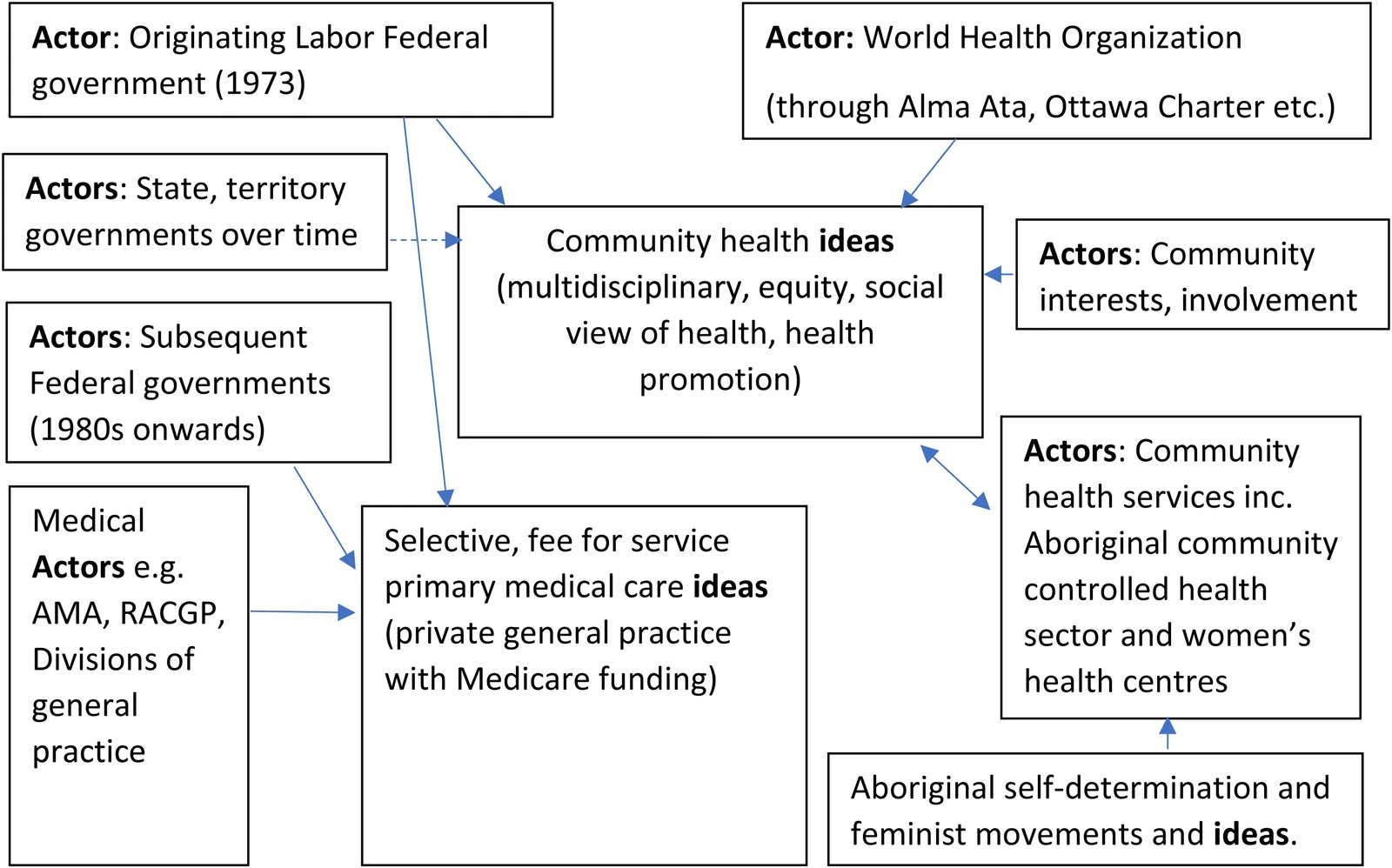
The interaction between actors and ideas shaping community health ideas in Australian policy.

AMA = Australian Medical Association. RACGP = Royal Australian College of General Practitioners. The dotted line from state, territory governments over time to community health ideas indicates the variable extent of their support.

## Limitations

We reviewed over 50 years of community health written policy in Australia. Written policy only describes intent not actual implementation, and these can diverge considerably in practice.^27^ In our considerable collective experience of working and researching in the community health sector, some polices (like the 1973 Community Health Program national policy) gave a framework of principles and rationale and guided implementation. Other policies seem to have been mainly rhetorical, and their impact on practice harder to discern. Sometimes significant decisions can go unmentioned in policy, while at other times, policies have been reversed or overridden. Nevertheless, interrogating policy content provides insight into the ideas and interests driving policy decisions that shape the health care system over decades and can yield valuable insights to help advocate for a better, more equitable health system.

## Conclusion

Our analysis of Australian policies provides examples of how shifting ideas and interests have influenced the contest and evolution of community health and primary health care concepts in Australia. Australia's federal/state political structure and mix of private and public health care systems are major drivers of these contested interpretations. The way policy is framed is influential, but framings often go unchallenged, particularly when there are powerful interests and ideas that influence policy, such as medical power and medicalised understandings of health. This reduces debate over what is possible. PHC ideas are contested globally, and our findings suggest the need for countries to:

1. Examine their history to understand how ideas and interests have shaped understandings of PHC and current policies.
2. Clarify differences between these understandings and assess their consistency with the principles of comprehensive PHC. This is particularly relevant to debates on universal health coverage, and the role of PHC.^4^
3. Pursue whole of health system policies that attend to the role of hospitals, medical practice, PHC, and whole of government policies to address disease prevention and health promotion. Having policy that embraces a social view of health will help counter narrower biomedical approaches that have less scope to reduce disease and improve health through action on social determinants of health.

The Alma Ata Declaration ideal of PHC remains as relevant as ever to address increasing rates of chronic disease, global pandemics such as COVID-19, and rising health inequities.^18^ Pursuing comprehensive PHC remains a vital goal globally to improve population health and health equity. Discussion of universal health coverage alone cannot illuminate these different approaches to PHC, and so it is crucial to move beyond a sole focus on universal health coverage in global policy debates to include advocacy for and implementation of comprehensive models of PHC.

## Supplemental Material

sj-docx-1-joh-10.1177_27551938251411284 - Supplemental material for Australian Community Health and Primary Health Care Policies from 1970s to 2020s: A Policy Analysis of Changing Interpretations Supplemental material, sj-docx-1-joh-10.1177_27551938251411284 for Australian Community Health and Primary Health Care Policies from 1970s to 2020s: A Policy Analysis of Changing Interpretations by Helen van Eyk, Toby Freeman, Connie Musolino, Denise Fry, Colin McDougall, Tony McBride, Virginia Lewis and Fran Baum in International Journal of Social Determinants of Health and Health Services

sj-docx-2-joh-10.1177_27551938251411284 - Supplemental material for Australian Community Health and Primary Health Care Policies from 1970s to 2020s: A Policy Analysis of Changing Interpretations Supplemental material, sj-docx-2-joh-10.1177_27551938251411284 for Australian Community Health and Primary Health Care Policies from 1970s to 2020s: A Policy Analysis of Changing Interpretations by Helen van Eyk, Toby Freeman, Connie Musolino, Denise Fry, Colin McDougall, Tony McBride, Virginia Lewis and Fran Baum in International Journal of Social Determinants of Health and Health Services

sj-docx-3-joh-10.1177_27551938251411284 - Supplemental material for Australian Community Health and Primary Health Care Policies from 1970s to 2020s: A Policy Analysis of Changing Interpretations Supplemental material, sj-docx-3-joh-10.1177_27551938251411284 for Australian Community Health and Primary Health Care Policies from 1970s to 2020s: A Policy Analysis of Changing Interpretations by Helen van Eyk, Toby Freeman, Connie Musolino, Denise Fry, Colin McDougall, Tony McBride, Virginia Lewis and Fran Baum in International Journal of Social Determinants of Health and Health Services
