# Supplementary material for: Australian Community Health and Primary Health Care Policies from 1970s to 2020s: A Policy Analysis of Changing Interpretations
Source: Int J Soc Determinants Health Health Serv. 2026 Jan 16;56(4):566–79. doi: 10.1177/27551938251411284 (PMC13646415; doi:10.1177/27551938251411284)
Supplement: Supplementary file 1 [file sj-docx-1-joh-10.1177_27551938251411284.docx]

# Biographical Sketch instructions:

Submit in paragraph form, typed double-spaced, approximately 100 words in length, maximum 150 words.

Provide specific information:

- present position;
- past positions;
- academic degrees, with dates and names of institutions;
- important publications;
- research interests.
