# Supplementary material for: Australian Community Health and Primary Health Care Policies from 1970s to 2020s: A Policy Analysis of Changing Interpretations
Source: Int J Soc Determinants Health Health Serv. 2026 Jan 16;56(4):566–79. doi: 10.1177/27551938251411284 (PMC13646415; doi:10.1177/27551938251411284)
Supplement: Supplementary file 2 [file sj-docx-2-joh-10.1177_27551938251411284.docx]

**Table 1 Policy documents relevant to primary health care in Australia, New South Wales, South Australia and Victoria 1970 to 2020 (by year of release)**

| **Year** | **Jurisdiction** | **Policy title** |
| --- | --- | --- |
| **1970s** |  |  |
| 1973 | Australia | A community health program for Australia: report from the National Hospitals and Health Services Commission, Interim Committee, June 1973 |
| 1973 | SA | Health services in South Australia: Report of the Committee of Inquiry into Health Services in South Australia |
| 1976 | Australia | Review of the community health program: report from the Hospitals and Health Service Commission, March 1976 |
| 1977 | NSW | Community health: book no 1 – general concepts |
| 1977 | NSW | Community health: book no 2 – goals for community health services |
| 1977 | NSW | Community health: book no 3 – organisational objectives for generalist services |
| 1977 | NSW | Community health: book no 4 – organisational objectives for community health services for emotional, social and health related problems |
| 1977 | NSW | Community health: book no 5 – organisational objectives for services for mothers, babies, children and adolescents |
| 1977 | NSW | Community health: book no 6 – organisational objectives for services for the aged and chronically ill |
| **1980s** |  |  |
| 1980 | SA | Community health overview paper |
| 1984 | SA | Discussion paper on community health: the scope of community health services, policy issues and future directions |
| 1984 | SA | Report of the Working Party on a Women's Health Policy |
| 1985 | Vic | Ministerial review of community health May 1985 |
| 1985 | Vic | Why women’s health? – discussion paper |
| 1986 | Australia | Looking forward to better health: Volume 1 final report – Better Health Commission |
| 1986 | SA | Community health strategy |
| 1987 | Vic | Policy and program statement: a new focus for community health centres |
| 1987 | Vic | Why women’s health? – Victorian women respond |
| 1988 | Australia | National policy on women's health: a framework for change - a discussion paper for community comment and response, February 1988 |
| 1988 | SA | A social health strategy for South Australia |
| 1988 | SA | Primary health care in South Australia: a discussion paper |
| 1989 | Australia | Review of the community health program 1986 – Australian Community Health Association |
| 1989 | Australia | A national Aboriginal health strategy |
| 1989 | Australia | National women’s health policy – advancing women’s health in Australia |
| 1989 | SA | Primary health care policy statement |
| 1989 | SA | South Australia’s social justice strategy: building a brighter future |
| 1989 | SA | Women’s health centres of South Australia: 5 year strategic plan |
| **1990s** |  |  |
| 1990 | SA | Planning for the health of South Australians: goals, strategies and targets |
| 1991 | Australia | “You have your moments”: a report of funding of peak health and community organisations – Parliament of Australia |
| 1991 | SA | Area health service administration in South Australia |
| 1992 | Australia | National commitment to improved outcomes in the delivery of programs and services for Aboriginal peoples and Torres Strait Islanders |
| 1992 | Vic | Victorian health system review |
| 1992 | Vic | Victorian health system review: Ministerial statement |
| 1993 | SA | A planning and management strategy: Metropolitan Services Division |
| 1993 | SA | Strategic directions for primary health care in South Australia |
| 1994 | Australia | Towards a national health policy: a discussion paper |
| 1994 | SA | Aboriginal health – dreaming beyond 2000: our future is in our history – South Australian Aboriginal health policy and strategic framework November 1994 |
| 1999 | NSW | New South Wales Aboriginal health strategic plan |
| 1999 | NSW | Ensuring progress in Aboriginal health: a policy for the NSW health system |
| **2000s** |  |  |
| 2000 | NSW | Strategic framework to advance the health of women |
| 2000 | Vic | Healthy Victorians 2000: Victorian Health Promotion Foundation – conceptual and strategic plan to the year 2000 |
| 2001 | SA | Final report: metropolitan community health services review |
| 2002 | Australia | Women’s health outcomes framework |
| 2002 | Vic | Towards a community health policy framework: discussion paper |
| 2002 | Vic | Valuing Victorian women: Victorian women’s health and wellbeing strategy - policy statement and implementation framework 2002–2006 |
| 2002 | Vic | Women’s safety strategy: a policy framework |
| 2003 | Australia | National strategic framework for Aboriginal and Torres Strait Islander health |
| 2003 | SA | Better choices better health: final report of the South Australian Generational Health Review April 2003 |
| 2003 | SA | First steps forward: South Australian health reform |
| 2003 | SA | Primary health care policy statement 2003-2007 |
| 2004 | Australia | Social and emotional well being framework: a national strategic framework for Aboriginal and Torres Strait Islander peoples’ mental health and social and emotional well being 2004-2009 |
| 2004 | NSW | NSW Health and Equity Statement: in all fairness - increasing equity in health across NSW May 2004 |
| 2004 | SA | Chronic disease: prevention and management opportunities for South Australia – The evidence for change to an integrated system of prevention, early intervention and management of key chronic diseases and risk factors |
| 2004 | Vic | General practitioners in community health services: strategy development update |
| 2004 | Vic | Community health services: creating a healthier Victoria |
| 2005 | SA | South Australian women’s health policy |
| 2005 | SA | Department of Health priorities for action 2005-2006 |
| 2006 | NSW | Integrated primary and community health policy 2007 - 2012 |
| 2006 | Vic | Women’s Health Matters: From Policy to Practice - 10 Point Plan for Victorian Women’s Health 2006-2010 |
| 2007 | SA | South Australia’s health care plan 2007-2016 |
| 2007 | SA | GP Plus health care strategy August 2007 |
| 2007 | SA | Aboriginal health policy |
| 2008 | SA | SA Health strategic plan 2008-2010 |
| 2008 | Vic | Primary Health Branch: Towards a demand management framework for community health services |
| 2009 | Australia | A healthier future for all Australians: final report June 2009 |
| 2009 | Australia | Australia: the healthiest country by 2020 – national preventative health strategy – overview 30 June 2009 |
| 2009 | SA | Chronic disease action plan 2009-2018 |
| 2009 | Vic | 10 point plan for Victorian Women’s Health 2010-2014 |
| 2009 | Vic | Guide to developing an optimal business model for general practice in community health |
| **2010s** |  |  |
| 2010 | Australia | A national health and hospitals network for Australia’s future: delivering better health and better hospitals |
| 2010 | Australia | A national health and hospitals network for Australia’s future: delivering the reforms |
| 2010 | Australia | Building a 21st century primary health care system: Australia's first national primary health care strategy |
| 2010 | Australia | National women’s health policy |
| 2010 | NSW | Maternal & child health primary health care policy |
| 2010 | NSW | Womens Health Plan 2009-2011 |
| 2010 | SA | Aboriginal health care plan 2010-2016 |
| 2010 | SA | Agreement on South Australian Aboriginal health and wellbeing - between the State of South Australia, the Australian Government and the Aboriginal Health Council of South Australia Inc 2010 -2015 |
| 2010 | SA | South Australian women’s health action plan: initiatives for 2010 and 2011 |
| 2011 | Australia | National strategic framework for rural and remote health |
| 2011 | Australia | National Aboriginal and Torres Strait Islander health plan 2013-2023 |
| 2011 | NSW | Aboriginal family health strategy 2011–2016: responding to family violence in Aboriginal communities |
| 2011 | SA | A framework for comprehensive primary health care services for Aboriginal people |
| 2011 | SA | Primary prevention plan 2011-2016 |
| 2011 | Vic | Victorian public health and wellbeing plan 2011–2015 |
| 2012 | NSW | NSW Aboriginal health plan 2013-2023 |
| 2012 | NSW | Guidelines for developing HealthOne NSW services |
| 2012 | SA | Review of non-hospital based services |
| 2013 | Australia | National primary health care strategic framework April 2013 |
| 2013 | SA | South Australia: a better place to live - promoting and protecting our community’s health and wellbeing 2013 |
| 2014 | NSW | NSW state health plan: towards 2021 |
| 2015 | Australia | Implementation plan for the national Aboriginal and Torres Strait Islander health plan 2013–2023 |
| 2015 | Australia | Primary Health Networks (PHNs) and Aboriginal community controlled health organisations (ACCHOS) – guiding principles |
| 2015 | NSW | NSW Aboriginal health partnership agreement 2015-2025 |
| 2015 | SA | Delivering Transforming Health: our next steps |
| 2015 | Vic | Implementing the Victorian public health and wellbeing plan 2015–2019: Taking action – the first two years |
| 2015 | Vic | Community health integrated program guidelines: direction for the Community Health Program |
| 2015 | Vic | Care for people with chronic conditions: guidelines for the Community Health Program |
| 2015 | Vic | Child health services: guidelines for the Community Health Program |
| 2015 | Vic | Refugee and asylum seeker health services: guidelines for the Community Health Program |
| 2015 | Vic | Victorian public health and wellbeing plan 2015–2019 |
| 2016 | Vic | Health 2040: advancing health, access and care |
| 2016 | Vic | Health 2040: achievements and next steps |
| 2017 | Australia | National strategic framework for Aboriginal and Torres Strait Islander peoples’ mental health and social and emotional wellbeing 2017-2023 |
| 2017 | NSW | NSW Health statement of commitment |
| 2017 | SA | SA Health strategic plan 2017-2020 |
| 2017 | Vic | Balit Murrup: Aboriginal social and emotional wellbeing framework 2017–2027 |
| 2017 | Vic | Korin Korin Balit-Djak: Aboriginal health, wellbeing and safety strategic plan 2017–2027 |
| 2018 | Australia | National women’s health strategy 2020-2030 |
| 2018 | SA | State public health plan 2019-2024 |
| 2018 | Vic | Victorian Auditor General’s Office: Community health program audit |
| 2018 | Vic | Community services quality governance framework: safe, effective, connected and person-centred community services for everybody, every time |
| 2018 | Vic | Victorian public health and wellbeing plan 2015–2019: action plan update |
| 2018 | Vic | Victorian Aboriginal Affairs framework 2018-2023 |
| 2019 | Australia | Australia’s long term national health plan to build the world’s best health system August 2019 |
| 2019 | NSW | NSW Women’s Health Framework |
| 2019 | Vic | Victorian public health and wellbeing plan 2019–2023 |
| 2019 | Vic | Community Health Taskforce: report to Government |
| 2019 | Vic | Department of Health and Human Services strategic plan |
| **2020** |  |  |
| 2020 | Australia | National agreement on closing the gap July 2020 |
| 2020 | Australia | Consultation paper: development of the National Preventive Health Strategy |
| 2020 | SA | South Australian health and wellbeing strategy 2020-2025 |
| 2020 | Vic | Community health reform plan 2020-2024 |
