# Supplementary material for: Australian Community Health and Primary Health Care Policies from 1970s to 2020s: A Policy Analysis of Changing Interpretations
Source: Int J Soc Determinants Health Health Serv. 2026 Jan 16;56(4):566–79. doi: 10.1177/27551938251411284 (PMC13646415; doi:10.1177/27551938251411284)
Supplement: Supplementary file 3 [file sj-docx-3-joh-10.1177_27551938251411284.docx]

**Figure 2 Timeline of policy developments related to primary health care in Australia, New South Wales, South Australia and Victoria 1970 - 2020**

*Governments in power and changes of government are marked in italics*

**AUSTRALIA**

1971

- *Liberal/National Country Party Coalition government in power from 1951-1972. McMahon was PM from 1971-1972, preceded by Gorton (1968-1971).*

1972

- *Election of Whitlam ALP government*

1973

- Establishment of Hospital and Health Services Commission
- “A Community Health Program for Australia – [Report](https://nla.gov.au/nla.obj-1746198092/view?partId=nla.obj-1752060509) of the Interim Committee of the National Hospital and Health Services Commission”
- Establishment of Central Australian Aboriginal [Congress](https://www.caac.org.au/news/community-control-history/) Health Centre in Alice Springs in June 1973.

1974

- Establishment of Medibank
- Funding of women’s health centres under Community Health Program

1975

- *Election of the Fraser Liberal/National Party Coalition government*
- The first Central Australian Aboriginal Congress clinic was opened (1975/76)

1976

- “[Review](https://nla.gov.au/nla.obj-2150893953/view?partId=nla.obj-2156312240) of the Community Health Program, Commonwealth Dept of Health and the Hospitals and Health Services Commission”

1978

- An Aboriginal delegation travelled to Kazakhstan and influenced the WHO Alma-Ata Declaration on Primary Health Care with its community-controlled model
- WHO Alma-Ata [Declaration](https://www.who.int/docs/default-source/documents/almaata-declaration-en.pdf?sfvrsn=7b3c2167_2)

1981

- WHO Global Strategy for [Health for All by the Year 2000](https://www.who.int/publications/i/item/9241800038)
- Abolition of Medibank as a universal public health insurance scheme

1983

- *Election of the Hawke ALP government*

1984

- Introduction of Medicare universal health insurance scheme

1985

- Establishment of the Better Health Commission
- Second national women’s health conference “Women's Health in a Changing Society” held in Adelaide

1986

- WHO [Ottawa Charter for Health Promotion](https://www.who.int/publications/i/item/ottawa-charter-for-health-promotion)
- “Review of the Community Health Program by ACHA” (Australian Community Health Association)
- Looking Forward to Better Health – report of Better Health Commission ([Hansard](https://parlinfo.aph.gov.au/parlInfo/search/display/display.w3p;db=CHAMBER;id=chamber/hansardr/1986-10-10/0035;query=Id:%22chamber/hansardr/1986-10-10/0035%22))

1987

- The Health Targets and Implementation (Health For All) Committee of AHMAC established with membership drawn from government health authorities at Commonwealth and State/Territory levels, the Consumers' Health Forum, and the Australian Institute of Health (AIH)
- ACHA funded by Cwlth to implement CHASP (Community Health Accreditation and Standards Program) across Australia
- Dept of Aboriginal Affairs approved funding for Congress Alukura program – a community-based program of antenatal, childbirth and postnatal care for Aboriginal women

1988

- 2^nd^ WHO International Conference on Health Promotion held in Adelaide
- Health for all Australians – report of Health Targets and Implementation Committee
- Establishment of the National Better Health Program
- “National policy on women’s health: a framework for change – a discussion paper for community comment and response”
- Report of National Aboriginal Health Strategy Working Party called for better coordination between Cwlth, states and Aboriginal community-controlled organisations

1989

- “National Women’s Health Policy: Advancing Women’s Health in Australia” ([summary](https://awhn.org.au/wp-content/uploads/2015/03/38_NationalWomensHealthPolicysummary.pdf))
- “National Aboriginal Health Strategy”

1990

- Establishment of National Health Strategy 1990-1993
- General Practice Evaluation Program funded

1991

- *Keating replaced Hawke as PM of ALP government*
- National program for the early detection of breast cancer
- National policy on the screening for the prevention of cervical cancer
- Introduction of General Practice Reform Strategy
- “You have your moments: a report of funding of peak health and community organisations”

1992

- NACCHO (National Aboriginal Community Controlled Health Organisations) established as the new national AMS (Aboriginal Medical Service) umbrella organisation replacing the National Aboriginal and Islander Health Organisation
- 1992-93 – the Australian Government committed funding for the establishment of the Divisions of General Practice

1994

- “Towards a National Health Policy – a discussion paper” (Health Ministers’ Forum 1994)
- Commencement of the Ambulatory Care Reform Program
- Release of Better Health Outcomes for Australians report

1995

- Commonwealth Dept of Health funded Co-ordinated Care Trials as a major health reform.

1996

- *Election of the Howard Liberal/National Party Coalition government*

1997

- Responsibility for provision of women’s health services handed to states although Cwlth continued to contribute funding through National Public Health Partnership

1998

- Review of the General Practice Strategy
- De-funding of the Australian Community Health Association

1999

- The Enhanced Primary Care (EPC) MBS (Medical Benefits Scheme) items introduced in 1999-2000 to improve the health and quality of life of older Australians, people with chronic conditions and those with multi-disciplinary care needs.
- Primary Health Care Access Program announced in 1999-2000 Cwlth Budget aimed at improving access to primary health care by facilitating increased community control, reforming existing health system structures, and increasing available resources within selected sites

2000

- [Introduction](https://www.aph.gov.au/Parliamentary_Business/Bills_Legislation/bd/Bd9900/2000bd013) of ‘Lifetime Health Cover’
- Introduction of Primary Health Care Research Evaluation and Development (PHCRED) Strategy

2002

- “Women’s health outcomes framework”

2003

- Review of Divisions of General Practice
- “National strategic framework for Aboriginal and Torres Strait Islander health”

2004

- MBS items introduced for a limited range of services provided by practice nurses when acting for a GP
- MBS rebates for a range of allied health and dental services introduced for patients with chronic conditions and complex care needs being managed by their GP under a multi-disciplinary care plan.
- A range of bulk billing incentive items introduced to encourage GPs to bulk bill concession card holders and children aged under 16.
- COAG agreed to national framework of [principles](https://www1.health.gov.au/internet/publications/publishing.nsf/Content/oatsih_implementationplan2007-2013~appendix3) for government service delivery to Indigenous Australians to provide a common framework between governments promoting flexibility and tailored responses to form partnerships with Indigenous communities
- “Social and emotional well being framework: a national strategic framework for Aboriginal and Torres Strait Islander peoples’ mental health and social and emotional well being 2004-2009”

2005

- [Chronic Disease Management](https://www1.health.gov.au/internet/main/publishing.nsf/Content/mbsprimarycare-chronicdiseasemanagement) (CDM) items were introduced to replace EPC care planning items. The CDM items were developed to better enable GPs to manage the health care of patients with chronic medical conditions, including patients who need multi-disciplinary care
- Rebate for most GP services increased from 85% to 100% of the Medicare schedule fee

2006

- National Chronic Disease [Strategy](https://www.health.qld.gov.au/__data/assets/pdf_file/0023/431609/nat-chronic-dis-strategy.pdf)
- Australian Better Health [Initiative](http://ncp.ncc.gov.au/docs/Council%20of%20Australian%20Governments%20Meeting%20-%2010%20February%202006.pdf)

2007

- *Election of Rudd ALP government*
- Launch of Close the Gap campaign

2008

- [WHO Commission](https://www.who.int/social_determinants/final_report/csdh_finalreport_2008.pdf) on the Social Determinants of Health
- National Health and Hospitals Reform Commission formed
- GP Super Clinics: Better health care for Australians. [National program guide](https://www1.health.gov.au/internet/main/publishing.nsf/Content/1879709D1C65D435CA257BF0001AFEE8/$File/National%20Program%20Guide%202010.pdf)
- National Preventive Health Taskforce
- National Partnership Agreement on Preventive [Health](https://www.federalfinancialrelations.gov.au/content/npa/health/_archive/health_preventive_national_partnership.pdf)

2009

- “A Healthier Future for all Australians: National Health and Hospitals Reform Commission [final report](https://apo.org.au/sites/default/files/resource-files/2009-07/apo-nid17921.pdf)”
- “Australia: The Healthiest Country by 2020 - [National Preventive Health Strategy](https://www.health.qld.gov.au/__data/assets/pdf_file/0022/424426/nphs-overview.pdf)”
- Release of [Social Justice 2008](https://humanrights.gov.au/our-work/aboriginal-and-torres-strait-islander-social-justice/publications/social-justice-report-2) report focused on human rights protection for Indigenous peoples; remote Indigenous education; Indigenous healing; and the progress on achieving Indigenous health equality

2010

- *Gillard replaced Rudd as PM of ALP government*
- *ALP forms minority government under Gillard with support of 3 independents and 1 Green*
- National Health and Hospitals Network [Agreement](https://www.federalfinancialrelations.gov.au/content/npa/health/_archive/national-agreement.pdf)
- GP Plus Super [Clinics](https://www1.health.gov.au/internet/main/publishing.nsf/Content/1879709D1C65D435CA257BF0001AFEE8/$File/National%20Program%20Guide%202010.pdf)
- “A National Health and Hospitals Network for Australia’s Future: Delivering better health and better [hospitals](C://Users/vane0006/AppData/Local/Downloads/NHHN%20-%20for%20Australia's%20future%20(1).pdf)”
- “Building a 21^st^ Century primary health care system: Australia’s first national primary health care [strategy](https://extranet.who.int/nutrition/gina/sites/default/filesstore/AUS%202010%20Building%20a%2021st%20Century%20Primary%20Health%20Care%20System%2C%20Australia%27s%20First%20National%20Primary%20Health%20Care%20Strategy.pdf)”
- [Announcement](https://pmtranscripts.pmc.gov.au/release/transcript-17444) of National Preventive Health Agency
- “National women’s health policy”

2011

- National Health Reform [Agreement](https://www.federalfinancialrelations.gov.au/content/npa/health/_archive/national-agreement.pdf)
- Medicare Locals replaced Divisions of General Practice
- Australian National Preventive Health Agency established under [legislation](https://www.legislation.gov.au/Details/C2010A00134) passed in 2010
- “National strategic framework for rural and remote health plan”

2013

- *Rudd replaced Gillard as PM of ALP Government*
- “National Primary Health Care Strategic Framework”
- “National Aboriginal and Torres Strait Islander Health [Plan](https://www1.health.gov.au/internet/main/publishing.nsf/content/b92e980680486c3bca257bf0001baf01/$file/health-plan.pdf) 2013-2023”
- *Election of Abbott Liberal/National Party Coalition government*

2014

- [Review of Medicare Locals](https://www1.health.gov.au/internet/main/publishing.nsf/content/A69978FAABB1225ECA257CD3001810B7/$File/Review-of-Medicare-Locals-may2014.pdf) by Prof John Horvath reinforced value of intermediary health organisations (aimed at improving primary health system)
- Withdrawal of National Partnership Agreement on Preventive Health budget
- National Preventive Health Agency [abolished](https://www.aph.gov.au/Parliamentary_Business/Bills_Legislation/Bills_Search_Results/Result?bId=r5203)

2015

- *Turnbull replaced Abbott as PM of Liberal/National Party Coalition government*
- Replacement of Medicare Locals with PHNs
- [Health Care Homes](https://www.aph.gov.au/About_Parliament/Parliamentary_Departments/Parliamentary_Library/FlagPost/2018/May/Health_care_homes) trial launched
- “PHN ACCHO Guiding [principles](https://www1.health.gov.au/internet/main/publishing.nsf/Content/F7AE823951279103CA257FEF000414C8/$File/PHN%20and%20ACCHO%20-%20Guiding%20Principles.pdf)”
- “[Implementation plan](https://www.health.gov.au/sites/default/files/documents/2020/12/implementation-plan-for-the-national-aboriginal-and-torres-strait-islander-health-plan-2013-2023.pdf) for the National Aboriginal and Torres Strait Islander Health Plan 2013-2023”

2016

- Aboriginal and Torres Strait Islander leaders issued the Redfern [Statement](https://www.reconciliation.org.au/wp-content/uploads/2017/11/The-Redfern-Statement.pdf), calling for Aboriginal and Torres Strait Islander autonomy and community control

2017

- “National strategic framework for Aboriginal and Torres Strait Islander peoples’ mental health and social and emotional wellbeing 2017-2023”

2018

- *Morrison replaced Turnbull as PM of Liberal/National Party Coalition government*
- “NACCHO [National Framework](https://ahcsa.org.au/resources/national-framework-continuous-quality-improvement-primary-health-care-aboriginal-torres-strait-islander-people-2018-2023/) for Continuous Quality Improvement in Primary Health Care for Aboriginal and Torres Strait Islander people, 2018-2023”
- “National women’s health strategy 2020-2030”

2019

- *Election of Morrison Liberal/National Party Coalition government*
- “Australia’s long term [national health plan](https://www.health.gov.au/sites/default/files/australia-s-long-term-national-health-plan_0.pdf) to build the world’s best health system”

2020

- COVID-19 pandemic declared by WHO
- “National Preventive Health [Strategy](https://consultations.health.gov.au/national-preventive-health-taskforce/consultation-paper-for-the-national-preventive-hea/supporting_documents/NPHS%20Consultation%20Paper%20%20PDF.pdf)”
- “New National [Agreement](https://www.closingthegap.gov.au/sites/default/files/files/national-agreement-ctg.pdf) on Closing the Gap”

**NEW SOUTH WALES**

1971

- *Askin Liberal government in power from 1965-1975*
- Redfern Aboriginal Medical Service, a community-controlled health service established in Redfern, Sydney

1974

- Opening of Leichhardt Women’s Community Health Centre – first women’s health centre in Australia

1975

- *Lewis replaced Askin as Liberal Premier*
- Opening of Liverpool Women’s Community Health

1976

- *Willis replaced Lewis as Liberal Premier*
- *Wran ALP government elected. Wran was Premier from 1976-1986*
- Commencement of nurse practitioner training to provide women’s health promotion services including reproductive health services
- Establishment of well women’s clinics by Family Planning Australia
- Women’s health initiative – women’s health nurses were funded.

1977

- Community health “Blue books” providing guidelines for operation of community health services
- Opening of first workers’ health centre in Sydney

1980

- NSW Community Health Association established

1982

- Establishment of NSW Dept of Health
- 1982-83 initial development of Community Health Accreditation and Standards Program (CHASP) standards and review process
- Hausfeld Committee of Inquiry to investigate Area Health Boards

1983

- 4 pilot Area Health Services formed and 12 Area Health Services gazetted recognising existing area health management arrangements

1986

- *Unsworth replaced Wran as Premier of ALP government*
- NSW Community Health Association held its first Community Health Conference.

1987

- NSW Dept of Health commenced training of women’s health nurses in the community to provide mobile breast and cervical cancer screening services, health promotion and counselling services

1988

- *Greiner Liberal government elected*

1992

- *Fahey replaced Greiner as Liberal Premier*

1995

- *Carr ALP government elected*

1999

- “NSW Aboriginal Health Strategic Plan 1999-2004”
- “Ensuring progress in Aboriginal health: a policy for the NSW health system”

2000

- “Strategic Framework to Advance the Health of Women”

2002

- “Women’s Health Outcomes Framework”

2004

- “NSW Health and Equity [Statement](http://hiaconnect.edu.au/old/files/NSW_Health_and_Equity_Statement.pdf) – In all fairness: Increasing health and equity across NSW”

2005

- *Iemma ALP government elected*

2006

- “Integrated Primary and Community Health Policy 2007-2012”

2008

- *Rees replaced Iemma as Premier of ALP government*

2009

- *Keneally replaced Rees as Premier of ALP government*

2010

- “Maternal & Child Health Primary Health Care [Policy](https://flinders-my.sharepoint.com/personal/muso0024_flinders_edu_au/Documents/ARC%20History%20of%20Community%20Health/Policy%20and%20archival/Policy%20docs/NSW%20Maternal%20&%20Child%20Health%20Primary%20Health%20Care%20Policy%202010.pdf)”
- Womens Health Plan 2009-2011

2011

- *O’Farrell Liberal government elected*
- Local Health Districts replaced Area Health Services
- “Aboriginal family health [strategy](https://www.health.nsw.gov.au/aboriginal/Publications/pub-family.pdf) 2011-2016”

2012

- “[Guidelines](https://www.health.nsw.gov.au/healthone/Documents/honswguidedevelop_11.pdf) for Developing HealthOne NSW Services”
- “NSW Aboriginal Health [Plan](https://www1.health.nsw.gov.au/pds/ActivePDSDocuments/PD2012_066.pdf) 2013-2023”

2014

- *Baird replaced O’Farrell as Liberal Premier*
- “NSW state health plan: towards 2021”

2015

- “NSW Aboriginal health partnership agreement 2015-2025”

2017

- *Berejiklian replaced Baird as Liberal Premier*

2019

- “NSW Women’s health framework”

**SOUTH AUSTRALIA**

1970

- *Dunstan ALP government elected. In power from 1970-1979*

1973

- “Health Services in South Australia: Report of the Committee of Enquiry into Health Services in South Australia”

1975

- Opening of Hindmarsh Women’s Health Centre with funding from national Community Health Program

1976

- Establishment of Foundation for Multidisciplinary Education in Community Health (FMECH) as an initiative of the Faculty of Medicine, University of Adelaide and SA Institute of Technology

1979

- *Corcoran replaced Dunstan as ALP Premier*
- *Tonkin Liberal government elected*
- Funding for Hindmarsh Women’s Health Centre ceased in 1979-80

1980

- Opening of Adelaide Women’s Health Centre
- “Community health overview paper”

1981

- Establishment of SA Community Health Association

1982

- *Bannon ALP government elected*

1983

- Women’s health centres opened in Elizabeth and Christies Beach
- Establishment of Nganampa Health Council

1984

- “Discussion paper on community health: The scope of community health services, policy issues and future directions”
- “Report of the Working Party on a Women’s Health Policy”
- Opening of Dale St Women’s Health Centre in Port Adelaide

1985

- Incorporation of Noarlunga Health Service – the largest community health service in SA

1986

- Establishment of Social Health Office
- “Community health strategy”

1987

- Publication of Uwankara Palyanyku Kanyintjaku report – ‘UPK: A strategy for well-being’ on health status of Aboriginal people living in the area of the Nganampa Health Council

1988

- “A Social Health Strategy for South Australia”
- “Primary Health Care in South Australia: A discussion paper”
- Establishment of Foundation SA (the SA Sport Promotion, Cultural and Health Advancement Trust) using tobacco tax dollars

1989

- “Primary Health Care Policy Statement”
- “South Australia’s social justice strategy: building a brighter future”
- “Women’s health centres of South Australia: 5 year strategic plan

1990

- First Social Health Atlas of SA released
- “Planning for the health of South Australians: goals, strategies and targets

1991

- Collapse of the State Bank
- 3 country women’s health services established under National Women’s Health Program and Policy
- “Area health service administration in South Australia”

1992

- *Arnold replaced Bannon as ALP Premier*

1993

- *Brown Liberal government elected*
- “Strategic Directions for Primary Health Care in South Australia”
- “A planning and management strategy: Metropolitan Services Division”

1994

- “Aboriginal health – dreaming beyond 2000: our future is in our history – South Australian Aboriginal health policy and strategic framework November 1994”

1996

- *Olsen replaced Brown as Liberal Premier*

1997

- *Olsen Liberal government re-elected*

2001

- *Kerin replaced Olsen as Liberal Premier*
- “Final report: metropolitan community health services review”

2002

- *Rann ALP government elected*

2003

- “Primary Health Care Policy Statement 2003-2007”
- “Better Choices Better Health: Final report of the South Australian Generational Health [Review](https://www.sahealth.sa.gov.au/wps/wcm/connect/f2f26480428ddf2ab41fb6e7eece1070/generationalhealthreviewreport-ce-0304.pdf?MOD=AJPERES&CACHEID=f2f26480428ddf2ab41fb6e7eece1070)” (a significant review that drove policy)
- “First steps forward” – SA Govt response to GHR

2004

- “Chronic Disease – Prevention & management opportunities for South Australia”

2005

- “South Australian women’s health policy”
- “Department of Health priorities for action 2005-2006”

2007

- “GP Plus Health Care Strategy”
- “South Australia’s Health Care [Plan](https://www.sahealth.sa.gov.au/wps/wcm/connect/893b1180428de54db565b7e7eece1070/sahcp-sahealth-20072016.pdf?MOD=AJPERES&amp;CACHEID=ROOTWORKSPACE-893b1180428de54db565b7e7eece1070-nwK2K-a) 2007-2016”
- “Aboriginal health policy”

2009

- “Chronic Disease Action Plan for South Australia 2009-2018”

2010

- “Aboriginal health care plan 2010-2016”
- “Agreement on South Australian Aboriginal health and wellbeing - between the State of South Australia, the Australian Government and the Aboriginal Health Council of South Australia Inc 2010 -2015”
- “South Australian women’s health action plan: initiatives for 2010 and 2011”

2011

- *Weatherill replaced Rann as ALP Premier*
- “Primary Prevention [Plan](https://www.sahealth.sa.gov.au/wps/wcm/connect/fd31550046cd63e9937dfb2e504170d4/PrimaryPreventionPlan20112016-SSS-HPB-1105.pdf?MOD=AJPERES&amp;CACHEID=ROOTWORKSPACE-fd31550046cd63e9937dfb2e504170d4-nwMlYCZ) 2011-2016”
- “A [Framework](https://www.sahealth.sa.gov.au/wps/wcm/connect/ac97b3804b5567bf88bafd9a087db869/PHCFramework-HSD-AH-120211.pdf?MOD=AJPERES&amp;CACHEID=ROOTWORKSPACE-ac97b3804b5567bf88bafd9a087db869-nwK3eR-) for Comprehensive Primary Health Care Services for Aboriginal People”
- All metropolitan publicly funded PHC services brought together in one centrally managed region

2012

- “[Review](http://www.cpsu.asn.au/upload/Campaign/nonhospital-report-PHCS-20121203.pdf) of Non-Hospital Based Services” (McCann review)

2013

- Cuts recommended in McCann review implemented
- “South Australia: a better place to live - promoting and protecting our community’s health and wellbeing 2013” – first state public health plan

2014

- Launch of Transforming Health focused on reforming SA’s hospital system

2015

- “Delivering Transforming Health: our next steps”

2018

- “State public health plan 2019-2024”
- *Marshall Liberal government elected*

2020

- “South Australian Health and Wellbeing [Strategy](https://www.sahealth.sa.gov.au/wps/wcm/connect/286484af-1cad-451d-a0c1-3b589a0b7485/Health+and+Wellbeing+Strategy+web+FINAL.pdf?MOD=AJPERES&amp;CACHEID=ROOTWORKSPACE-286484af-1cad-451d-a0c1-3b589a0b7485-nwLyszF) 2020-2025”

**VICTORIA**

1972

- *Hamer replaced Bolte as Liberal Premier. (Bolte had been in power since 1955)*
- Victorian Aboriginal Health Service established

1973

- Fitzroy Aboriginal Health Service established
- Formation of Melbourne Women’s Health Collective

1974

- Collingwood Women’s Health Centre established

1981

- *Thompson replaced Hamer as Liberal Premier*

1982

- *Cain ALP government elected*

1985

- “Ministerial Review of Community Health”
- “Why women’s health? – discussion paper”

1986

- 1986-87 budget – funds made available for first fully funded women’s health centre and Women’s Health Information Centre

1987

- Passage of Tobacco [Act](https://www.vichealth.vic.gov.au/~/media/About%20Us/Attachments/TobaccoAct1987.ashx) 1987 and founding of Victorian Health Promotion Foundation funded with state tobacco fees (VicHealth)
- “Policy and program statement: a new focus for community health centres”
- “Why women’s health? Victorian women respond”

1988

- Health Services [Act](http://classic.austlii.edu.au/au/legis/vic/consol_act/hsa1988161/) 1988 formally recognised and strengthened community management of Vic community health centres
- Opening of Women’s Health West, first women’s health centre in Vic

1990

- *Kirner replaced Cain as ALP Premier*
- First community-based cervical screening program established

1992

- *Kennett Liberal government elected. In power until 1999*
- “Victorian health system review”
- Defunded much community development work in health during the 1990s

1998

- “A Stronger Primary Health and Community Support System: Policy Directions”

1999

- *Bracks ALP government elected*

2000

- “Healthy Victorians 2000”

2002

- “Towards a community health policy framework – discussion paper”
- “Valuing Victorian women: Victorian women’s health and wellbeing strategy - policy statement and implementation framework 2002–2006”

2004

- “GPs in Community Health Services Strategy”
- “Community health services: creating a healthier Victoria”

2006

“Women’s Health Matters: From Policy to Practice - 10 Point Plan for Victorian Women’s Health 2006-2010”

2007

- *Brumby replaced Bracks as ALP Premier*

2008

- “Towards a demand management framework for community health services”
- “Demand management framework”

2009

- “Guide for an optimal model for general practice in community health”

2010

- *Baillieu Liberal government elected*
- “Victorian public health and wellbeing plan 2011-2015”

2013

- *Napthine replaced Baillieu as Liberal Premier*

2014

- *Andrews ALP government elected*

2015

- “Community Health Integrated Program Guidelines: Direction for the Community Health [Program](https://www2.health.vic.gov.au/primary-and-community-health/community-health/community-health-program/chip-guidelines)” (re-issued 2019)
- “Implementing the Victorian public health and wellbeing plan 2015–2019: Taking action – the first two years”

2016

- “Health 2040”
- “Care for People with Chronic Conditions: Guidelines for the Community Health [Program](https://www2.health.vic.gov.au/about/publications/policiesandguidelines/chronic-care-guide-and-case-studies)” (re-issued 2019)

2017

- “Balit Murrup: Aboriginal social and emotional wellbeing framework 2017–2027”
- “Korin Korin Balit-Djak: Aboriginal health, wellbeing and safety strategic plan 2017–2027”

2018

- “Victorian Auditor General’s Office: Community health program audit”
- “Community services quality governance framework: safe, effective, connected and person-centred community services for everybody, every time”
- “Victorian public health and wellbeing plan 2015–2019: action plan update”
- “Victorian Aboriginal Affairs framework 2018-2023”

2019

- “Victorian public health and wellbeing plan 2019–2023”
- “Community Health Taskforce: report to Government”

2020

- “Community Health Reform [Plan](https://www2.health.vic.gov.au/about/publications/policiesandguidelines/community-health-reform-plan) 2020-2024”
